# A Rare Case of Congenital Left Ventricular Diverticulum With Apical Thrombus: Diagnostic Challenges and Management Insights

**DOI:** 10.7759/cureus.56662

**Published:** 2024-03-21

**Authors:** Isaac Alsallamin, Eman N Ahmed, Maha Wazir, Sameer M Mtour

**Affiliations:** 1 Clinical Medicine, Northeast Ohio Medical University, Cleveland, USA; 2 Internal Medicine, St. Vincent Charity Medical Center, Cleveland, USA; 3 Internal Medicine, Case Western Reserve University School of Medicine, Cleveland, USA; 4 Internal Medicine, University Hospitals Cleveland Medical Center, Cleveland, USA; 5 Internal Medicine, Alfaisal University College of Medicine, Riyadh, SAU; 6 Medicine, Khyber Teaching Hospital, Peshawar, PAK; 7 Cardiology, Al-Makassed Hospital, Jerusalem, PSE

**Keywords:** intracardiac thrombus, cardiac mri, left ventricular diverticulum, ventricular diverticulum, stroke

## Abstract

A myocardial diverticulum is a rare congenital anomaly characterized by pouch-like protrusions within the myocardial wall, which can potentially lead to various cardiac complications. This case report describes a unique presentation of a left ventricular diverticulum (LVD) with an associated apical thrombus, highlighting the diagnostic and management challenges posed by this condition. A 58-year-old man presented to the emergency department with left arm weakness, wrist drop, and chest pain, initially raising concerns for a stroke. Diagnostic evaluations, including echocardiography and magnetic resonance imaging (MRI), revealed a small focal outpouching at the left ventricular apex, consistent with a congenital LVD containing a thrombus. This diagnosis was supported by the patient’s historical imaging dating back to 2007, which had similarly identified this outpouching. The patient was managed with anticoagulation therapy, transitioning from heparin to warfarin, alongside standard cardiac care. This case underscores the importance of considering myocardial diverticulum in the differential diagnosis of patients presenting with cardiac symptoms that might initially suggest more common conditions, such as stroke. It also highlights the essential role of echocardiography and MRI in diagnosing and managing myocardial diverticula.

## Introduction

A myocardial diverticulum, often found in the left ventricle, is an outpouching that includes all myocardial layers and differs from an aneurysm due to its ability to contract synchronously. The phenomenon of congenital left ventricular diverticulum (LVD) has been extensively studied in pediatric populations and is recognized as a developmental anomaly occurring during the fourth week of embryogenesis [1]. Ohlow has noted that this anomaly develops from defective, fragile tissue transforming into a sponge-like mass amidst the myocardium’s trabeculae, eventually forming a diverticulum [1]. The diverticula’s frequent apical positioning can be attributed to the halted development of the primitive para-midline mesoderm between the 14th and 18th days of embryogenesis, following the differentiation of the parietal and ventral parts. These rare diverticula vary in size, ranging from 0.5 cm to significantly larger sizes of 8 cm to 9 cm [1]. Classifications of these outpouchings may be based on pathology or imaging characteristics. Diagnosis typically begins with cardiac echocardiography, with computed tomography (CT) scans and magnetic resonance imaging (MRI) providing further details; however, confirmatory diagnosis through pathology is often not necessary. The presentation of LVD is usually asymptomatic, discovered incidentally during evaluations for cardiothoracic issues, comprehensive chest pain assessments, or follow-ups in high-risk cardiac patients. We present a case of a 58-year-old man with a previously undiagnosed congenital LVD, revealed through a workup for chest pain and left arm weakness.

## Case presentation

A 58-year-old man presented to the emergency department reporting left arm pain and weakness, wrist drop, and chest pain. He described the left arm pain as mild, with preserved sensation. The chest pain was characterized as dull, generalized, constant, and non-radiating. Upon examination in the observation unit, he was fully oriented and had an unremarkable cardiac examination. Neurological examination highlighted intact sensation, markedly reduced strength, and range of motion on the left side, power 2/5, with hyperreflexia, a claw-like deformity of the left hand, and an unsteady gait.

His medical history included hypertension, hyperlipidemia, chronic obstructive pulmonary disease, gastroesophageal reflux disease, surgical release for cubital tunnel syndrome in the left arm, Raynaud’s phenomenon affecting the left hand, and alcohol use disorder with no history of alcohol withdrawal or delirium tremens. He was adherent to his medications, which included amlodipine (10 mg daily), hydrochlorothiazide (25 mg daily), baby aspirin, atorvastatin 40 mg daily, a multivitamin, and a pantoprazole 40 mg daily.

**Figure 1:**
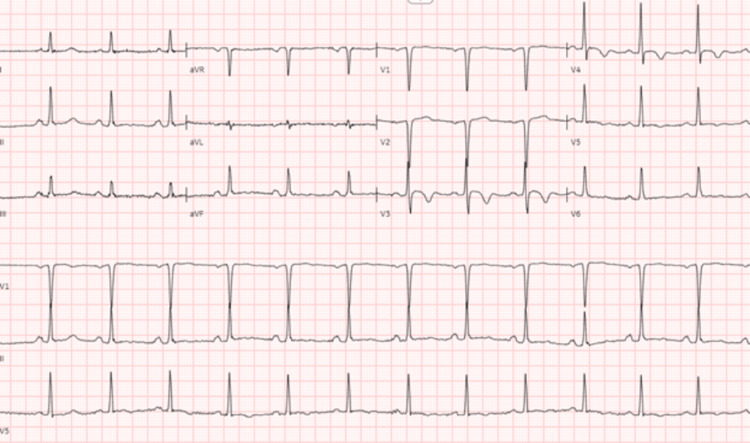
ECG on arrival to ED. There are no new ST-T changes or arrhythmias (Figure 1), persistent old changes, lost T wave inversion V1-V2, T wave inversion V3-V4, and flat T wave V5-6, with signs of LVH noticed in previous ECGs. ECG, electrocardiogram; ED, emergency department

The initial differential diagnosis considered acute stroke, acute coronary syndrome, cervical nerve compression, and left radial nerve palsy due to compression. Diagnostic evaluations, including serial electrocardiograms (ECGs), were negative for new ST-T changes or arrhythmias (Figure 1), recalled persistent old changes, lost T wave inversion V1-V2, T wave inversion V3-V4, and flat T wave V5-6, with signs of LVH noticed in previous hospitalization.

**Figure 2:**
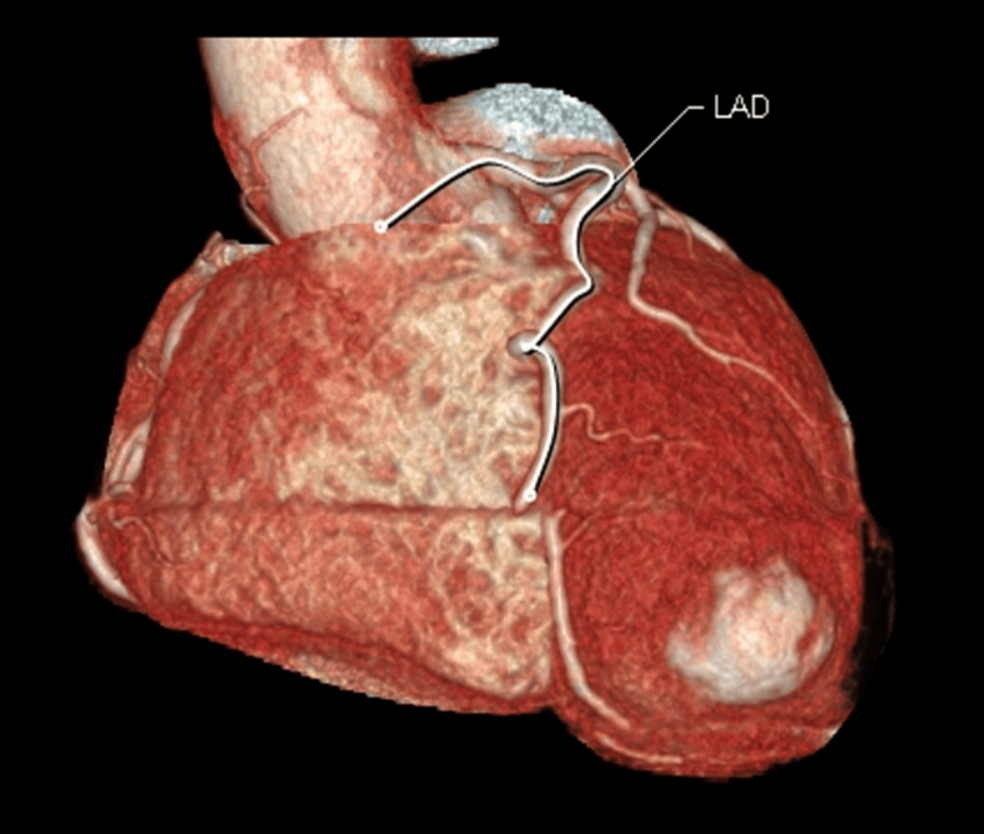
Coronary CTA showing LAD. No significant findings suggestive of significant obstruction in the coronary arteries, LM, LAD, LCX, and RCA. This image shows the CTA only of the LAD. CTA, computed tomography angiogram; LAD, left anterior descending artery

Cardiac enzymes were within reference limits. Coronary CT angiography (CTA) revealed normal coronary anatomy without atherosclerotic changes, radiologically very low suspicion for obstructed coronaries with a calcium score of zero (Figure 2).

**Figure 3:**
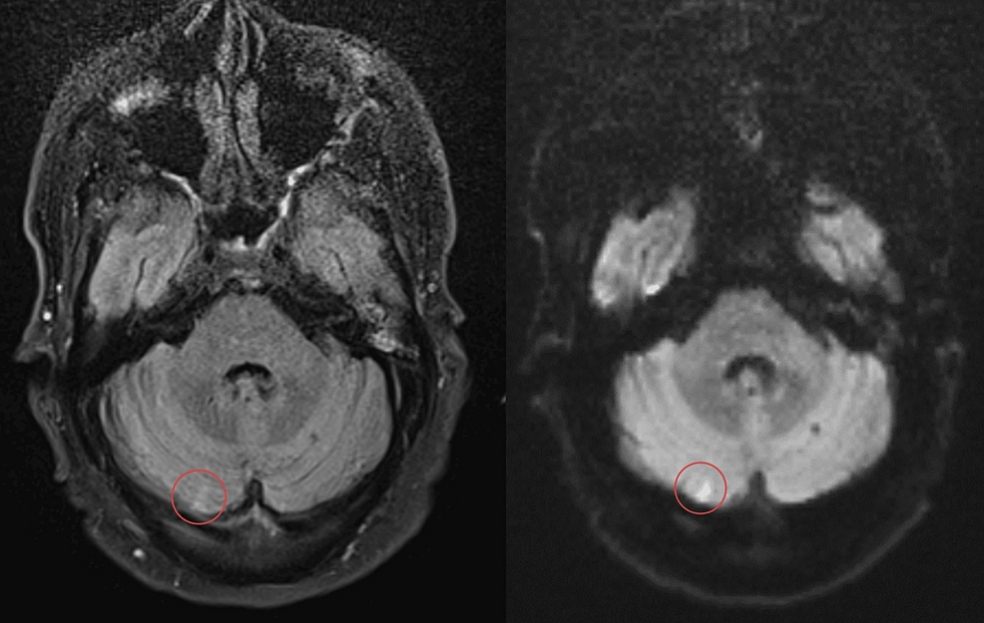
Brain MRI. Brain MRI showing a small focus of diffusion restriction within the right cerebellar lobe that likely represents a focus of acute/early subacute infarct, with no significant mass effect. MRI, magnetic resonance imaging

Given the negative ECG for new changes, negative serial cardiac enzymes, and very low suspicious CTA for obstructive CAD, we were confident to report the results as no significant coronary obstruction and rule out acute coronary syndrome. Alcohol levels trend down from 200 mg/dL to 153 mg/dL. The MRI of the cervical spine and brain identified a small subacute cerebellar stroke (Figure 3), deemed unrelated to his symptoms, and no significant cervical spine stenosis was observed.

**Figure 4:**
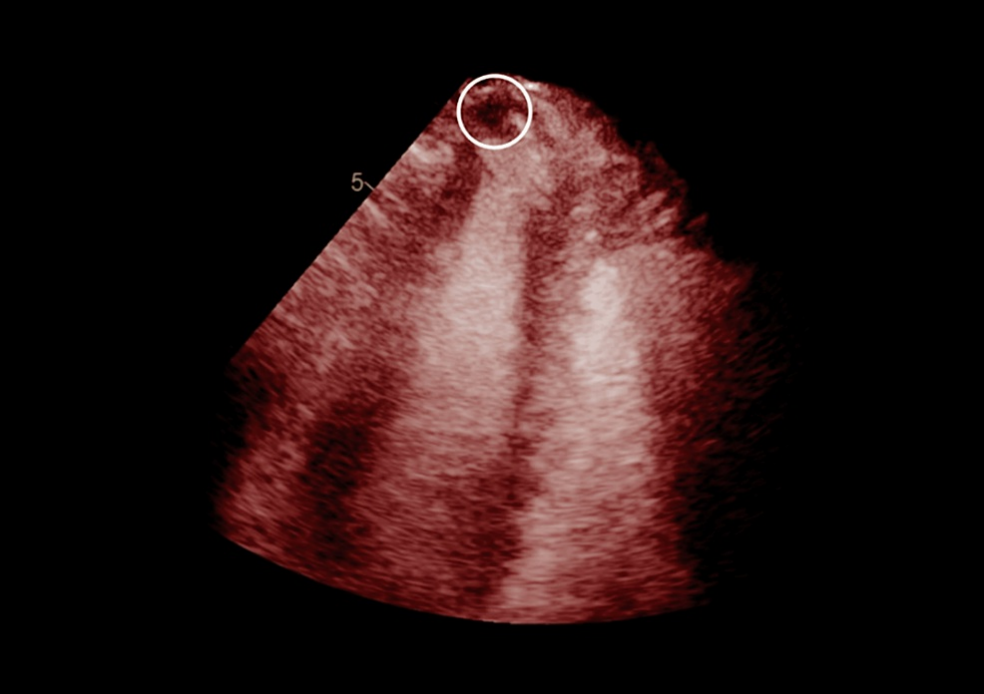
Apical thrombus, echogenic mass suspected for an apical clot. A moderately sized fixed LV thrombus is noted in the apex. The LV thrombus measures approximately 10 mm by 10 mm. LV, left ventricle

An echocardiogram revealed a suspicious apical thrombus and left ventricular apical akinesia, and there was no evidence of a patent foramen ovale (Figure 4), prompting further investigation.

**Figure 5:**
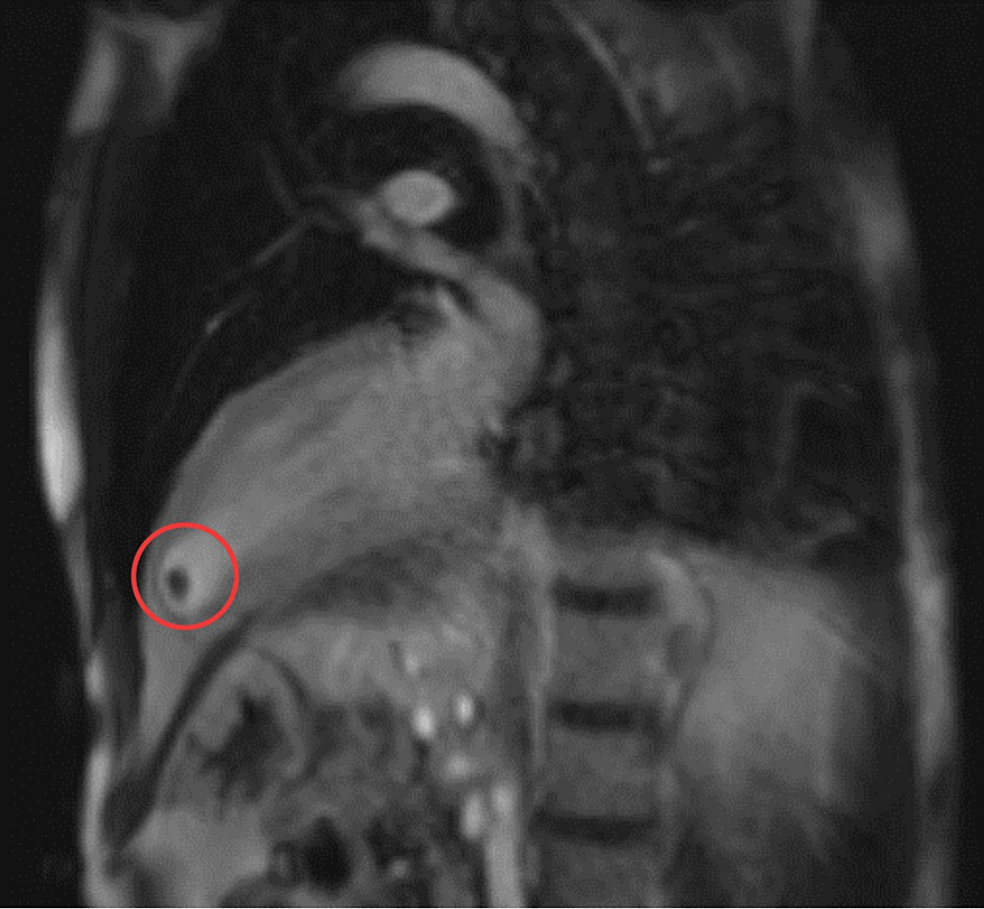
Cardiac MRI showing a small focal outpouching within the left ventricular apex, likely present since a CT exam from 2007. Cardiac MRI before contraction, The non-mobile mass is likely a thrombus. This finding most likely represents a congenital LVD. Within this diverticulum, there is a thrombus. MRI, magnetic resonance imaging; CT, computed tomography; LVD, left ventricular diverticulum (the poor image quality is due to some motion artifact)

**Figure 6:**
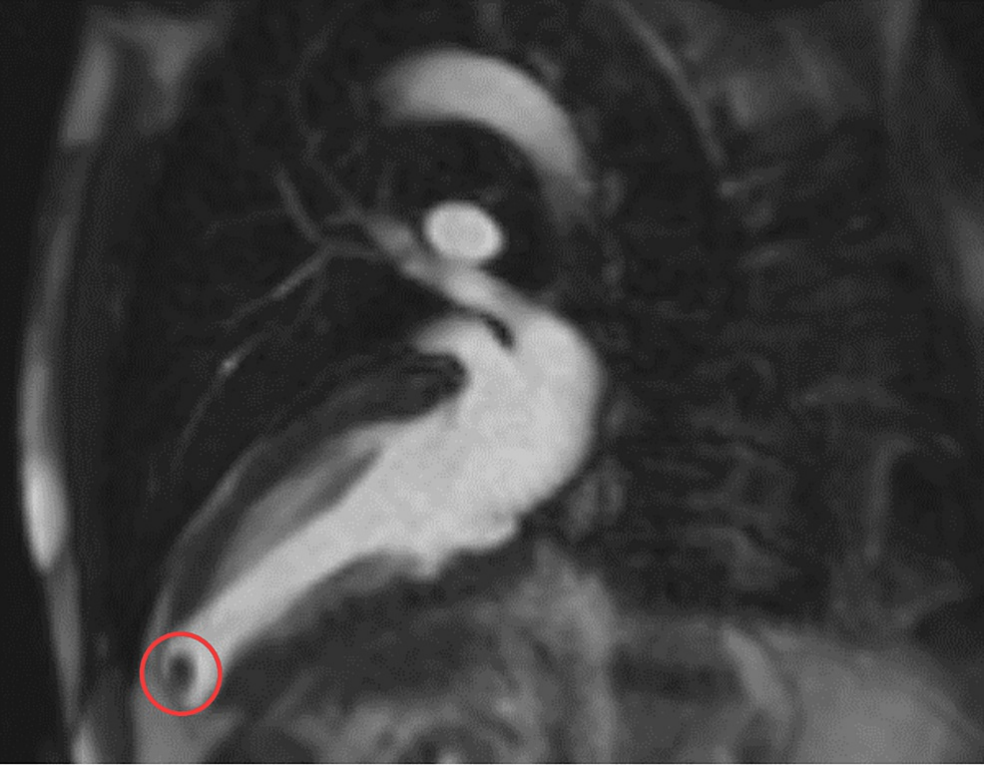
Cardiac MRI during systole. The non-mobile mass is likely a thrombus. MRI, magnetic resonance imaging (poor quality of the image due to some motion artifact)

Follow-up with a transesophageal echocardiogram confirmed a thrombus within an LVD. Subsequent cardiac MRI identified a small focal outpouching at the left ventricular apex, findings consistent with a CT exam from 2007, thereby suggesting the presence of a congenital LVD with an associated thrombus (Figures 5-6).

The patient was initiated on anticoagulation therapy, transitioning from heparin to warfarin, and continued aspirin and statin upon discharge. He was scheduled for an event monitor and follow-up imaging, including a head CT and cardiac MRI, with outpatient visits to cardiology and neurology.

Follow-up visits after two weeks, three months, and five months showed progressive improvement in his symptoms. The patient reported an improvement in weakness and pain, with ongoing but improving weakness in his left hand, allowing him to lift it without difficulty. He experienced some balance issues but no falls. Examination revealed improved muscle strength, assessed using the Medical Research Council scale, where 0 indicates no muscle contraction and 5 represents normal strength. The left triceps showed full strength (5/5), arm flexion was near normal (4/5), wrist extension was weak (2/5), finger abduction was moderately weak (3/5), and finger extension showed severe weakness (1/5). Left hip flexion was almost normal but slightly reduced (5-/5), indicating a strength just below normal, and foot dorsiflexion was better than moderate weakness but not fully normal (4+/5). The patient had intact cranial nerves and a wide-based gait, indicating a stable yet cautious walking pattern due to balance issues.

## Discussion

This case report discusses a rare occurrence of LVD presenting with apical thrombus, highlighted by a 58-year-old patient’s unique presentation of left arm weakness, wrist drop, and chest pain. These symptoms initially raised concerns for stroke, directing initial evaluations toward neurological rather than cardiac anomalies. However, echocardiographic findings followed by cardiac MRI confirmed the diagnosis of LVD, underscoring the complexity of diagnosing this condition.

Recent studies utilizing multi-detector CTA have reported an increased LVD prevalence of 2.2%, likely due to enhanced detection rates among patients with chest pain or known cardiac disorders [2]. LVD shows a higher incidence in Asian and European populations, predominantly affecting younger women and those with a genetic predisposition [3-5]. Associated anomalies include Cantrell’s syndrome and hypertrophic cardiomyopathy, among others, with a significant risk of cardiac death (5% within 2.5 years), often due to rupture [3-7]. LVD is categorized based on its location (intra-abdominal or intrathoracic) and the myocardial wall’s pathological features (muscular or fibrous) [1-9].

Differentiating LVD from left ventricular aneurysms and pseudo-aneurysms is critical, achieved primarily through imaging techniques like cardiac MRI, which offers detailed insights into the diverticulum’s structure and function [10-12]. Clinical presentations of LVD are varied, ranging from asymptomatic cases to those with thromboembolic events or heart failure [3,11,12].

Another differential diagnosis is cardiac non-compaction, which has a very unique cardiac echo and MRI findings of spongy myocardium, spongiform cardiomyopathy, and hypertrabeculation.

Diagnostic strategies emphasize using echocardiography, CT, and MRI to provide comprehensive views of the heart’s structure and function. Notably, cardiac MRI serves as a cornerstone for confirming LVD, offering precise information on myocardial edema, fibrous or necrotic tissues, and the viability of myocardial revascularization [2,13-15]. Despite the availability of various diagnostic tools, the absence of standardized treatment guidelines necessitates a tailored approach to management based on individual patient characteristics [1].

Therapeutic strategies for LVD are determined on a case-by-case basis, with small, asymptomatic cases often managed conservatively. Medical management includes not only using aspirin, beta-blockers, and statins for associated myocardial ischemia but also anticoagulation to treat and prevent thrombus formation [1,16]. In cases of large hypo/akinetic aneurysms or following systemic embolization, anticoagulation with warfarin is recommended [1,16]. Surgical intervention may be considered for symptomatic patients or those at risk of serious complications, such as malignant ventricular arrhythmias or cardioembolic events [17].

Treatment is case-by-case or individualized if the patient is asymptomatic, treated with conservative management and observation and follow-up cardiac echo. If the patient is symptomatic, then they will be treated accordingly. If the patient has signs of CAD, then they will be treated with revascularization, optimizing medical therapy using aspirin, statin, beta-blockers, and angiotensin-converting enzyme inhibitors. For ventricular arrhythmia, beta blockers are used initially, and for recurrent ventricular arrhythmia, several antiarrhythmic agents are used; the last option is ablation if persistent. Signs of heart failure due to LV dysfunction can be optimized with guideline-directed medical therapy (GDMT). Most patients with evidence of thrombus or thromboembolic phenomena will require anticoagulation. A surgical approach is limited and restricted for high-risk patients for wall rupture, length, and site or if the patient is going for any cardiac surgery especially if pseudoaneurysm is more suspected than diverticulum.

LVD can lead to a variety of complications, including thromboembolic events, infective endocarditis, and sudden cardiac death. IE was reported either isolated or associated with LVD pouch unexplained if related or was an incidental finding during workup for IE, possibly as a result of infected clots or direct infection. Sudden death could result from complications, such as massive embolization, fatal arrhythmia, or rupture LVD. The decision to surgically excise a diverticulum is often weighed against the high LVD risk for rupture, complications, uncontrolled disease, and potential risks of perioperative morbidity and mortality [1,17-19]. In our case, follow-up involved medical management, anticoagulation therapy, and ongoing outpatient monitoring.

## Conclusions

This case report underscores the critical importance of accurately diagnosing myocardial diverticulum, a rare congenital heart anomaly marked by pouch-like protrusions in the myocardial wall, and differentiating it from other cardiac outpouchings, such as aneurysms, pseudo-aneurysms, and double-chambered left ventricles. It highlights the essential role of echocardiography as a noninvasive and effective diagnostic tool complemented by MRI for confirmatory diagnosis, especially in distinguishing between muscular and fibrous types of diverticula for guiding treatment plans. This case also highlights the potential of advanced imaging techniques in facilitating accurate diagnoses without invasive procedures, advocating for further research to refine management strategies and improve outcomes for patients with this uncommon condition.
